# The Novel *Cucurbitaceae* miRNA *ClmiR86* Is Involved in Grafting-Enhanced Phosphate Utilization and Phosphate Starvation Tolerance in Watermelon

**DOI:** 10.3390/plants10102133

**Published:** 2021-10-08

**Authors:** Weifang Wu, Haoshun Zhao, Qin Deng, Haiyang Yang, Xiaoxiao Guan, Rui Qi, Pibiao Shi, Jinghua Yang, Mingfang Zhang, Zhongyuan Hu

**Affiliations:** 1 Laboratory of Germplasm Innovation and Molecular Breeding, Institute of Vegetable Science, Zhejiang University, Hangzhou 310058, China; 21416055@zju.edu.cn (W.W.); 22016057@zju.edu.cn (H.Z.); 21516069@zju.edu.cn (Q.D.); 21916162@zju.edu.cn (H.Y.); 22016161@zju.edu.cn (X.G.); 22016167@zju.edu.cn (R.Q.); 21316062@zju.edu.cn (P.S.); yangjinghua@zju.edu.cn (J.Y.); 2 Hainan Institute of Zhejiang University, Sanya 572025, China; 3 Key laboratory of Horticultural Plant Growth, Development & Quality Improvement, Ministry of Agriculture, Hangzhou 310058, China

**Keywords:** phosphate starvation response, phosphate utilization, grafting, watermelon

## Abstract

Watermelon (*Citrullus lanatus*) is a globally important Cucurbitaceae crop in which grafting is commonly used to improve stress tolerance and enhance nutrient utilization. However, the mechanism underlying grafting-enhanced nutrient assimilation remains unclear. Here, we demonstrate the possible involvement of a novel *Cucurbitaceae* miRNA, *ClmiR86*, in grafting-enhanced phosphate-starvation tolerance via *CALCINEURIN B-LIKE INTERACTING PROTEIN KINASE 5* (*ClCIPK5*) suppression in watermelon. Transcript analyses revealed that the induction of *ClmiR86* expression was correlated with the downregulation of *ClCIPK5* in squash-grafted watermelon under phosphate starvation. In addition, the differential expression of *ClmiR86* in various watermelon genotypes was consistent with their phosphate utilization efficiency. Furthermore, *ClmiR86* overexpression in Arabidopsis enhanced root growth and phosphate uptake under phosphate starvation and promoted inflorescence elongation under normal conditions. These results suggest that the *ClmiR86–ClCIPK5* axis is involved in phosphate starvation response as well as grafting-enhanced growth vigor and phosphate assimilation. The present study provides valuable insights for investigating long-distance signaling and nutrient utilization in plants.

## 1. Introduction

Grafting—the unification of different parts of two or more plants that grow together and form a new plant [1]—was found to improve resistance to various biotic and tolerance of various abiotic stresses [2,3,4,5]. Watermelon (*Citrullus lanatus*), a popular fresh fruit, is an economically important cash crop grown globally. Grafted watermelons using squash (*Cucurbita moschata*) and bottle gourd (*Lagenaria siceraria*) as the rootstocks were first adopted in Japan in the late 1920s [6,7]. Since then, grafting has been commonly used in watermelon production to improve disease resistance, specifically against the soil-borne Fusarium wilt disease. In addition, squash grafting was found to increase nutrient uptake in watermelon [8,9,10,11,12].

Phosphorus is a crucial and limiting soil nutrient, and it is fundamental to plant growth, development, and propagation [13,14]. However, because of precipitation and mineralization, the only form of soil phosphorus that can be assimilated by plants (inorganic phosphate) for vegetative growth and development is usually scarce [13,15,16]. As a result, plants have evolved several adaptive responses, such as the expansion of the root system to accelerate soil exploration [17,18], improvement of high-affinity phosphate uptake capacity [19], and production and secretion of organic acids and phosphatases to solubilize and mobilize phosphate in the soil and apoplast [15,20].

miRNAs are 20–24-nucleotide single-stranded RNA molecules that inhibit gene expression at the post-transcriptional level. Several miRNAs play critical roles in diverse physiological phenomena, such as growth, development, organogenesis, and biotic and abiotic stress response [21,22,23,24]. Specifically, some miRNAs have been identified as the key players in phosphate uptake and transport in plants, such as phosphate starvation-induced *miR399, miR827,* and *miR2111* [25,26,27]. In addition, phosphate starvation regulated *miR156, miR778, miR828, miR169, miR395*, and *miR398* [25,26]. The miR399 (a–f) family was first reported to negatively regulate the expression of *PHOSPHATE OVER ACCUMULATOR2* (*PHO2*), thereby enhancing phosphate translocation from roots to shoots [28,29,30,31]. *miR399* species also acted as graft-transmissible signals under phosphate starvation, and mature *miR399* was highly accumulated in OXmiR399/WT chimera roots, while the corresponding primary transcripts were absent in wildtype roots [25].

Calcineurin B-like (CBL) protein and CBL-interacting protein kinase (CIPK) complexes are well-known components of the Ca^2+^ signaling networks, which allow plants to fine-tune their responses to various stresses, such as salinity; cold; drought; and potassium, nitrate, and phosphate deficiency; these complexes are also involved in abscisic acid (ABA) signaling under stress [32,33,34,35,36]. For instance, CIPK3 acts as a cross-talk component between cold stress and ABA signaling [37] by physically and functionally interacting with CBL9 and forming a specific complex that functions in ABA response during seed germination [38,39]. The CBL10–CIPK24 pathway was essential for salinity tolerance by regulating Na^+^ homeostasis in *Arabidopsis* [40]. CIPK23, activated by binding with CBL1 and CBL9, regulated leaf transpiration and enhanced K^+^ uptake under potassium deficiency by phosphorylating the K^+^ transporter [33,41,42]. A recent study revealed that CBL1–CIPK23 and CBL9–CIPK23 complexes also phosphorylated the nitrate transporter (CHL1) to maintain a low-level primary response to nitrate deficiency [43]. In *Brassica napus, BnCIPK6* was strongly induced by phosphate deficiency, and it functionally interacted with BnCBL1. Ectopic *BnCIPK6* expression in *Arabidopsis* enhanced growth and biomass production under phosphate deficiency. Therefore, the BnCBL1–BnCIPK6 axis is involved in plant response to and tolerance of phosphate starvation [36]. To date, however, possible mechanisms underlying the involvement of CIPKs in phosphate starvation response and the post-transcriptional regulation of these kinases remain unknown.

Previously, we reported that phosphate uptake and utilization efficiency of squash-grafted watermelons were higher than those of self-rooted watermelons under phosphate starvation [9]. However, we did not explore the molecular effect of squash root on the phosphate starvation response of watermelon. To this end, in the present study, we demonstrated the possible involvement of a novel grafting-induced watermelon miRNA, *ClmiR86*, in phosphate accumulation. We also explored the expression profile of *ClmiR86* and its respective targets (*ClCIPK5/AtCIPK5/AtCIPK5*) under phosphate starvation in watermelon and transgenic *Arabidopsis*. Gene expression analysis revealed that *ClmiR86* likely regulates the phosphate starvation response of squash-grafted watermelon leaves by suppressing *ClCIPK5.* Furthermore, *ClmiR86* may be related to enhanced phosphate utilization efficiency (PUE) in watermelon. Ectopic *ClmiR86* expression in *Arabidopsis* enhanced root growth under phosphate starvation and growth vigor during the reproductive period. Our findings provide important evidence of the role of *ClmiR86* in grafting-enhanced phosphate utilization and phosphate starvation tolerance in watermelon.

## 2. Results

### 2.1. ClmiR86 May Enhance PUE of Watermelon

**Figure 1:**
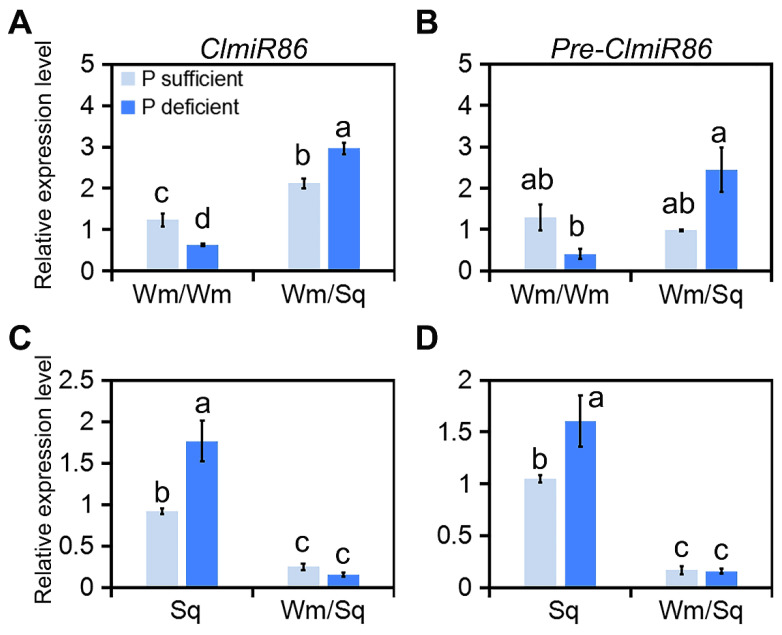
Grafting induced expression of *ClmiR86* and *Pre-ClmiR86* in watermelon leaves. Expression of (**A**) *ClmiR86* and (**B**) *Pre-ClmiR86* in the leaves of auto- and squash-grafted watermelon with or without P deficient treatment for 6 h. “Wm/Wm” and “Wm/Sq” represent auto-, and squash-grafted watermelon (Zaojia), respectively. Expression of (**C**) *ClmiR86* and (**D**) *Pre-ClmiR86* in the roots of non-grafted and grafted squash roots. “Sq” and “Wm/Sq” represent non-grafted and grafted squash roots, respectively. Different lower-case letters denote a significant difference in relative expression level (*p* < 0.05, one-way ANOVA and then Tukey’s test for multiple comparisons). Values are means ± SD (*n* = 3).

Our previous study demonstrated that under phosphate deficiency, phosphate uptake and PUE were significantly enhanced in squash-grafted watermelon compared with those in self-rooted watermelon [9]. Using small RNA deep sequencing of the same grafted watermelons, we identified *ClmiR86* as a candidate long-distance signal between the scion and rootstock, since it exhibited a reverse expression pattern in these tissues after grafting. A qRT-PCR analysis confirmed that squash grafting induced and decreased *ClmiR86* levels in watermelon leaves and squash roots, respectively (Figure 1A,C), indicating that *ClmiR86* may be transported from the rootstock to scion. Moreover, phosphate starvation further enhanced *ClmiR86* accumulation in squash-grafted watermelon leaves, but not in auto-grafted watermelon leaves (Figure 1A). Interestingly, *ClmiR86* was induced by phosphate starvation in non-grafted squash roots, but not in grafted rootstock (squash root; Figure 1C), and *ClmiR86* precursor showed a very similar expression profile to mature *ClmiR86* in grafted watermelon leaves and squash roots under both phosphate-sufficient and phosphate-deficient conditions (Figure 1B,D). These findings indicate that *ClmiR86* may act as a graft-transmissible signal (rootstock to scion) and be involved in the grafting-triggered phosphate starvation response.

**Figure 2:**
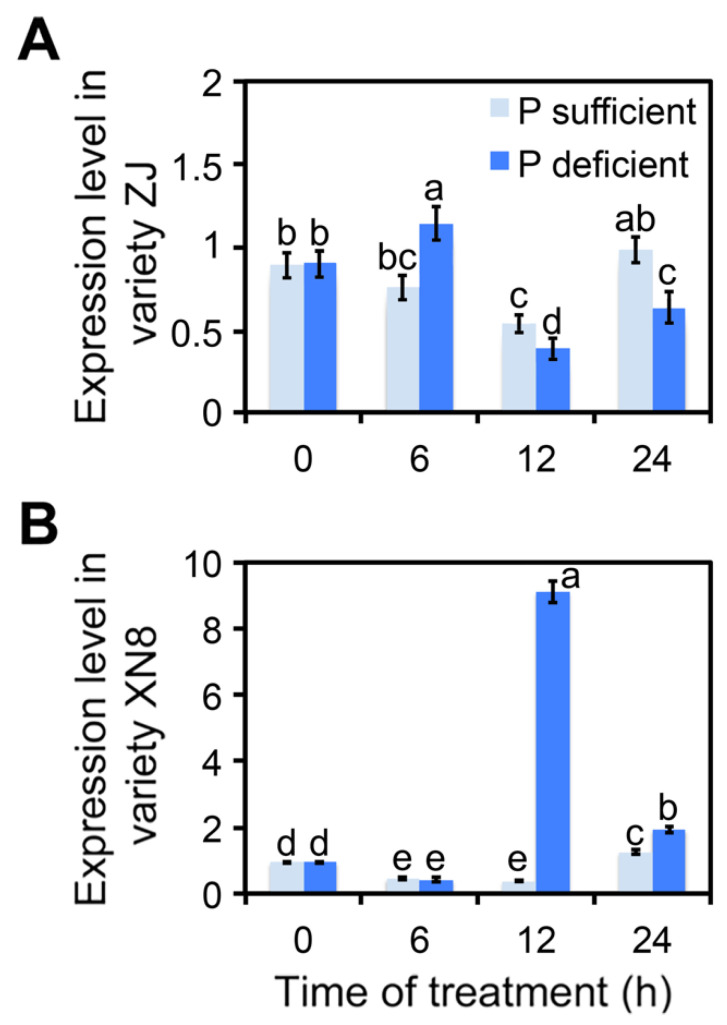
*ClmiR86* expression varies in roots of watermelon genotypes with different P utilization efficiencies. Expression of *ClmiR86* in the roots of (**A**) inefficient P utilization watermelon genotype “ZJ” and (**B**) efficient P utilization genotype “XN8” under different P concentrations. Different lower-case letters denote a significant difference in relative expression level (*p* < 0.05, one-way ANOVA and then Tukey’s test for multiple comparisons). Values are means ± SD (*n* = 3).

To investigate whether *ClmiR86* accumulation is consistent with improved phosphate utilization, we tested *ClmiR86* expression patterns in watermelon genotypes with different PUEs. In our previous study, ZJ, a low-PUE genotype, exhibited relatively less biomass under phosphate sufficiency and low PUE under phosphate deficiency [44]. A cyclic variation in *ClmiR86* expression with photoperiod was observed in both genotypes. Phosphate deficiency slightly and transitorily enhanced *ClmiR86* expression in ZJ (Figure 2A). Meanwhile, in the high-PUE genotype XN8, *ClmiR86* expression was significantly induced after 12 h of phosphate starvation (Figure 2B). This genotype-dependent response of *ClmiR86* expression to phosphate stress indicates its possible involvement in phosphate starvation response and utilization in watermelon.

### 2.2. ClmiR86 Suppresses ClCIPK5, AtCIPK5, and AtCIPK25 at the Post-Transcription Level

**Figure 3:**
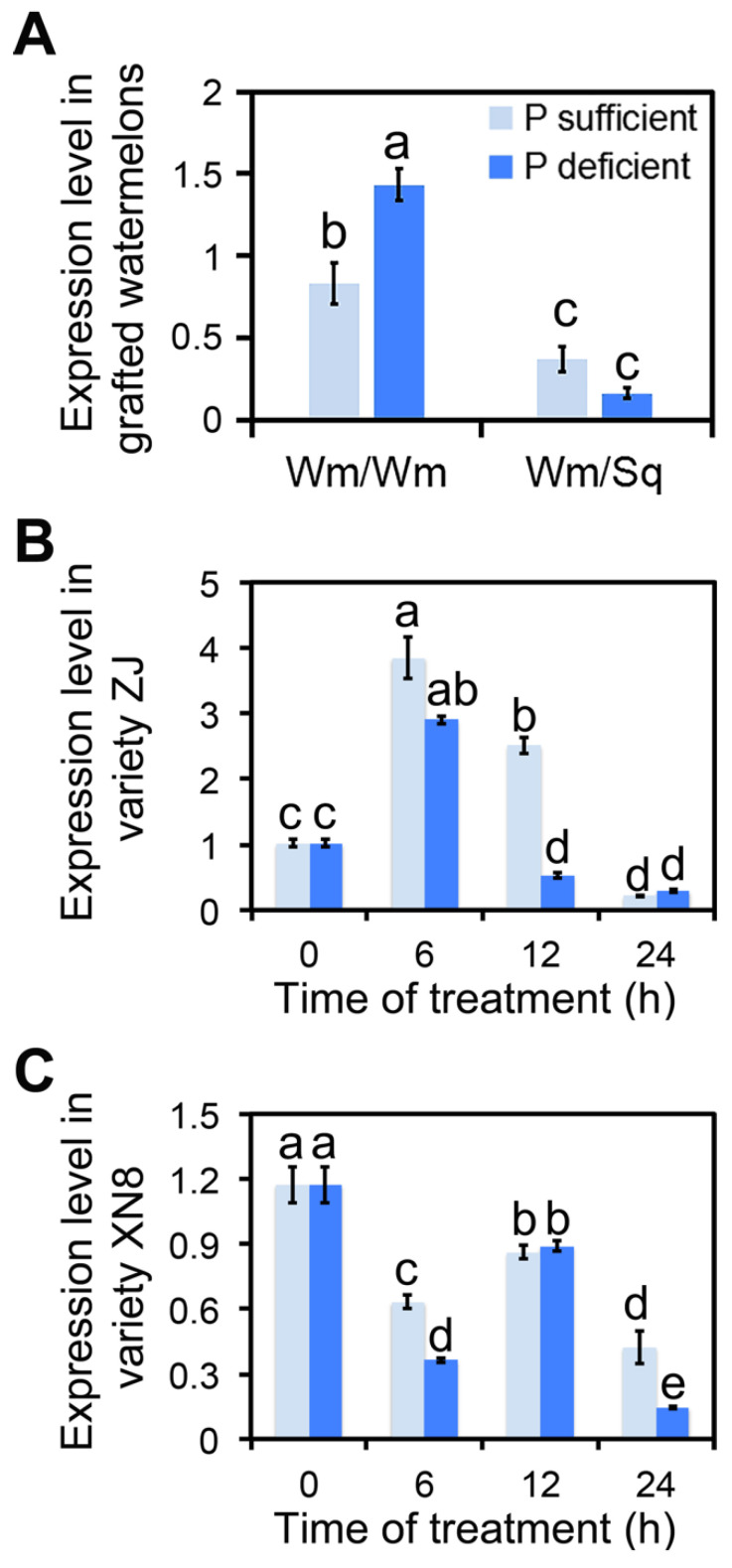
*ClCIPK5* was regulated by grafting and phosphate deficient stress. (**A**) Expression of *ClCIPK5* in the leaves of auto- and squash-grafted watermelon. “Wm/Wm” and “Wm/Sq” represent auto-, and squash-grafted watermelon (ZJ), respectively. (**B**) Expression of *ClCIPK5* in the roots of inefficient P utilization genotype “ZJ” under different P concentrations. (**C**) Expression of *ClCIPK5* in the roots of efficient P utilization genotype “XN8” under different P concentrations. Different lower-case letters indicate significant in expression level (*p* < 0.05, one-way ANOVA and then Tukey’s test for multiple comparisons). Values are means ± SD (*n* = 3).

miRNAs regulate the expression of target genes via binding mRNA transcripts, leading to mRNA degradation and/or translational repression. To better understand the biological functions of *ClmiR86* in watermelon, the effects of grafting and phosphate deficiency were examined on the expression of *ClCIPK5*, a putative target gene of *ClmiR86*. Squash-grafting suppressed *ClCIPK5* expression in watermelon leaves compared with auto-grafting. Moreover, phosphate deficiency dramatically enhanced *ClCIPK5* accumulation in auto-grafted watermelon leaves, but slightly inhibited its expression in squash-grafted watermelon leaves (Figure 3A). A similar cyclic variation in *ClCIPK5* expression was also observed in the two watermelon genotypes with different PUEs (Figure 3B,C). The negative correlation between *ClCIPK5* and *ClmiR86* expression under both grafting and phosphate deficiency indicates that squash-grafting might promote the phosphate starvation response of watermelon via the *ClmiR86*–*ClCIPK5* axis (Figure 1 and Figure 3).

**Figure 4:**
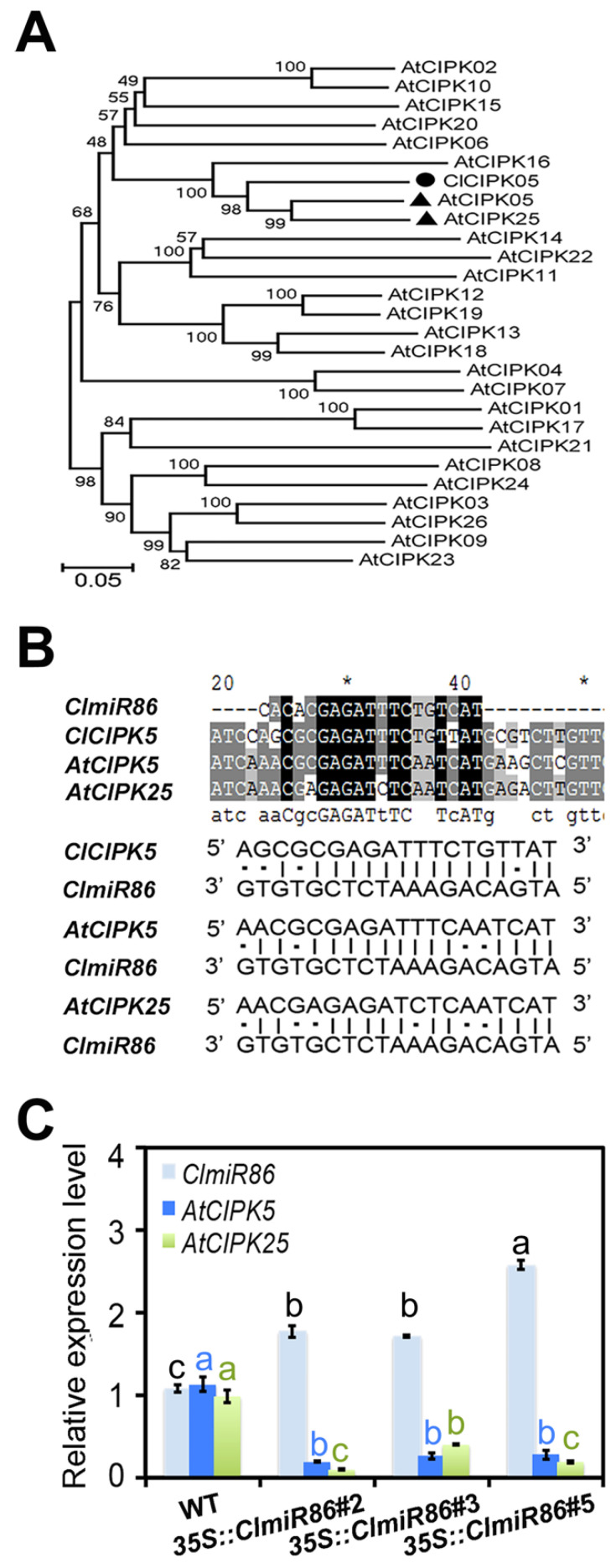
*ClmiR86* targets the transcription of *AtCIPK5* and *AtCIPK25* in transgenic *Arabidopsis.* (**A**) A Neighbour–Joining tree of 26 CIPK amino acid sequences from *Arabisopsis* and ClCIPK5. (**B**) The putative *ClmiR86* target site was conserved in these CIPKs. (**C**) The expression of *ClmiR86, AtCIPK5,* and *AtCIPK25* in three independent transgenic lines (*35S::ClmiR86*#2, #3, #5) over-expressing precursor of *ClmiR86* in *Arabidopsis* Col-0 background. Different lower-case letters with same font color denote a significant difference in relative expression level of each gene (*p* < 0.05, one-way ANOVA and then Tukey’s test for multiple comparisons). Values are means ± SD (*n* = 3). “*” was use to show the 30th and 50th cite of the sequence respectively.

To further verify the miRNA–target interaction, we also examined *AtCIPK5* and *AtCIPK25* expression in three independent transgenic lines (35S::*ClmiR86*#2, #3, and #5) overexpressing the *ClmiR86* precursor in the *Arabidopsis* Col-0 background. *AtCIPK5* and *AtCIPK25* were identified as the orthologous genes of *ClCIPK5* in *Arabidopsis* (Figure 4A), and the putative *ClmiR86* target site was also conserved in these two CIPKs (Figure 4B). qRT-PCR revealed a reduction in *AtCIPK5* and *AtCIPK25* expression in all p35S::*ClmiR86* lines (Figure 4C). Interestingly, *miR86* transcripts were detected in the wildtype, suggesting that this miRNA is present in *Arabidopsis*. These results indicate that *miR86* functions may be conserved between watermelon and *Arabidopsis* via the post-transcriptional regulation of CIPKs.

### 2.3. Ectopic ClmiR86 Expression Enhanced Phosphate Starvation Tolerance

**Figure 5:**
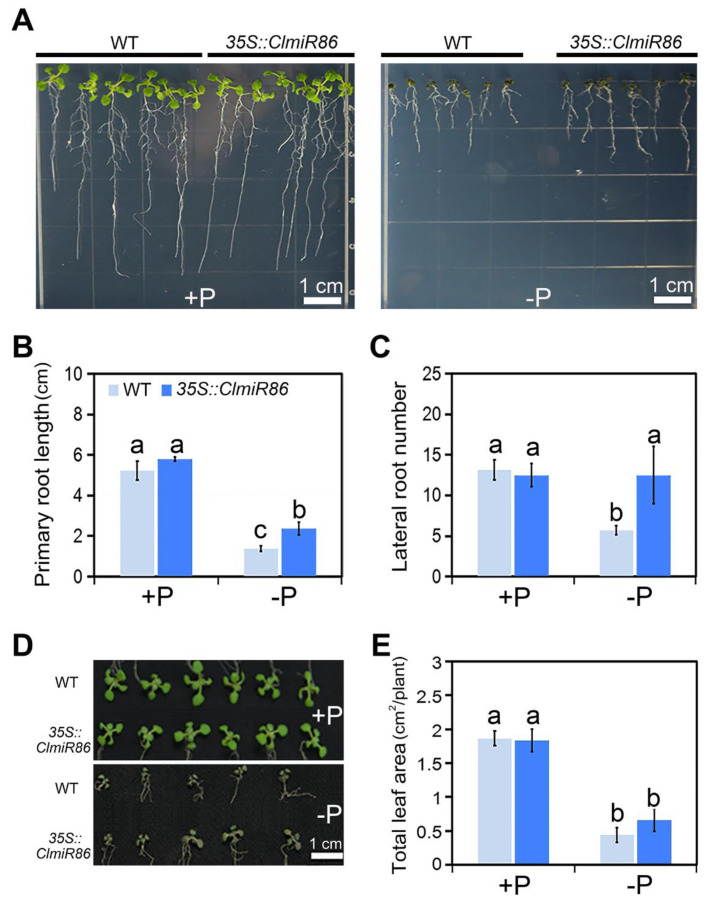
Ectopic expression of *ClmiR86* enhanced *Arabidopsis* root growth under P starvation at early stage. (**A**) Root phenotype of wild type (WT; Col-0) and *35S::ClmiR86 Arabidopsis* when grown on +P or –P MS medium for 7 days. (**B**) Primary root length of wild type and *35S::ClmiR86* seedlings when grown under P sufficient or deficient conditions. (**C**) Lateral root number of the wild type and *35S::ClmiR86* seedlings under P sufficient or deficient conditions. (**D**) Leaf phenotype of wild type (WT; Col-0) and *35S::ClmiR86 Arabidopsis* when grown on +P or –P MS medium for 7 days. (**E**) Leaf area of the wild type and *35S::miR86* seedlings submitted to P sufficient or deficient conditions. Different lower-case letters denote a significant difference in root growth or leaf area (*p* < 0.05, one-way ANOVA and then Tukey’s test for multiple comparisons). Values are means ± SD (*n* = 6).

To gain further insight into *ClmiR86* function in plants, wildtype and transgenic *Arabidopsis* lines overexpressing *ClmiR86* were examined under phosphate starvation. No obvious phenotype was identified in transgenic plants grown under phosphate sufficiency (Figure 5A). However, 35S::*ClmiR86* seedlings showed significantly increased primary root length after 1 week of phosphate starvation compared with the wildtype seedlings (Figure 5A,B). Furthermore, phosphate starvation significantly decreased lateral root number in the wildtype seedlings but did not decrease this number in 35S::*ClmiR86* seedlings (Figure 5A,C). Moreover, leaf growth in both wildtype and transgenic plants were dramatically inhibited by phosphate starvation, whereas ectopic *ClmiR86* expression slightly rescued leaf area under stress (Figure 5D,E). These results suggest that *ClmiR86* overexpression enhances root tolerance to phosphate starvation, and this effect may be associated with its regulation of CIPKs. Interestingly, *PHO2*, a well-known phosphate starvation-responsive target *of miR399*, was also downregulated in 35S::*ClmiR86* seedlings (Figure S1A). Moreover, *pho2* mutant seedlings showed a root response similar to 35S::*ClmiR86* seedlings (Figure 5 and Figure S1).

**Figure 6:**
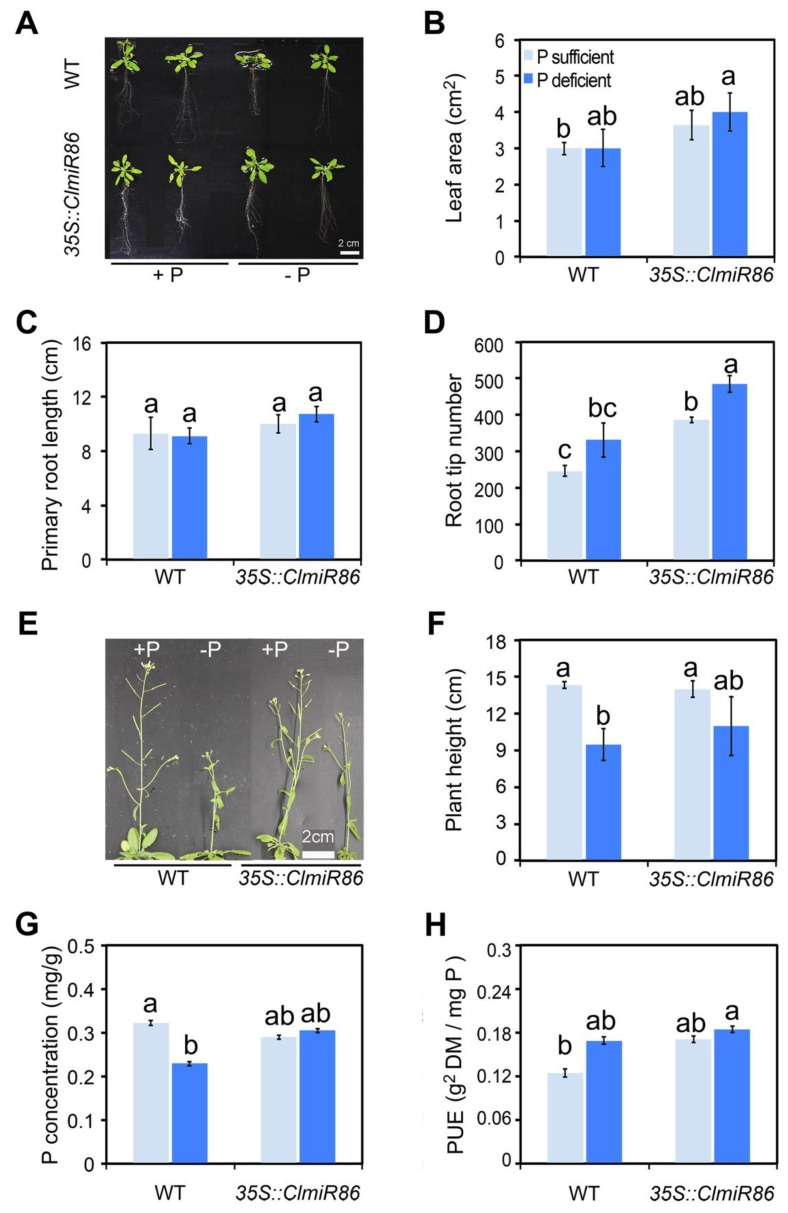
Phenotypes of *Arabidopsis* transgenic plants overexpressing *ClmiR86* under P starvation conditions at adult-plant stage. (**A**) *Arabidopsis* Seedlings of wild type (WT; Col-0) and *35S::ClmiR86* in WT backgrounds were grown on Jiffy seedling culture substrate for 22 days and then transferred to hydroponic culture with one-half Hoagland solution containing 0.01 mM P for low P treatment for additional 7 days. (**B**) Leaf area of the wild type and *35S::miR86* plants submitted to P sufficient or deficient conditions for 7 days. (**C**) Primary root length of wild type and *35S::ClmiR86* plants submitted to P sufficient or deficient conditions for 7 days. (**D**) Root tip number of the wild type and *35S::ClmiR86* plants submitted to P sufficient or deficient conditions for 7 days. (**E**) Wild type (WT) and *35S::miR86 Arabidopsis* adult-plants (22-d-old) were transferred to hydroponic culture with different P concentrations (0.3 or 0.01 mM P) and treated for 17 days. (**F**) Plant/inflorescence height of the wild type and *35S::miR86* plants submitted to P sufficient or deficient conditions for 17 days. (**G**) P concentration of the wild type and *35S::miR86* plants submitted to P sufficient or deficient conditions for 17 days. (**H**) PUE of the wild type and *35S::miR86* plants submitted to P sufficient or deficient conditions for 17 days. Different lower-case letters indicate significant difference in different plant growth parameters (*p* < 0.05, one-way ANOVA and then Tukey’s test for multiple comparisons). Values are means ± SD (*n* = 5).

To further verify the role of *ClmiR86* in phosphate starvation response, we examined the root and inflorescence phenotypes of transgenic *Arabidopsis* plants under phosphate starvation at the adult stage. After 1 week, 35S::*ClmiR86* plants grown under phosphate starvation showed significantly increased root tip number compared with plants grown under phosphate-sufficient conditions. Such differences were not observed in wildtype plants (Figure 6A,D). Phosphate deficiency did not affect leaf area or primary root length in any line (Figure 6B,C). When the phosphate starvation period was extended to 17 days, inflorescence length and height of wildtype plants were significantly reduced, although the flowering time remained similar among all lines. *ClmiR86* overexpression evidently relieved the inhibitory effects of phosphate starvation on inflorescence elongation (Figure 6E,F). Moreover, phosphate starvation significantly decreased the phosphate levels in wildtype plants, but not in transgenic plants (Figure 6G). In addition, wildtype seedlings showed slightly lower PUE than 35S::*ClmiR86* seedlings under phosphate sufficiency and phosphate deficiency slightly increased PUE (Figure 6H). These results suggest a pivotal role of *ClmiR86* in phosphate signaling during root and shoot development.

### 2.4. Ectopic ClmiR86 Expression Enhanced Plant Growth under Normal Conditions

**Figure 7:**
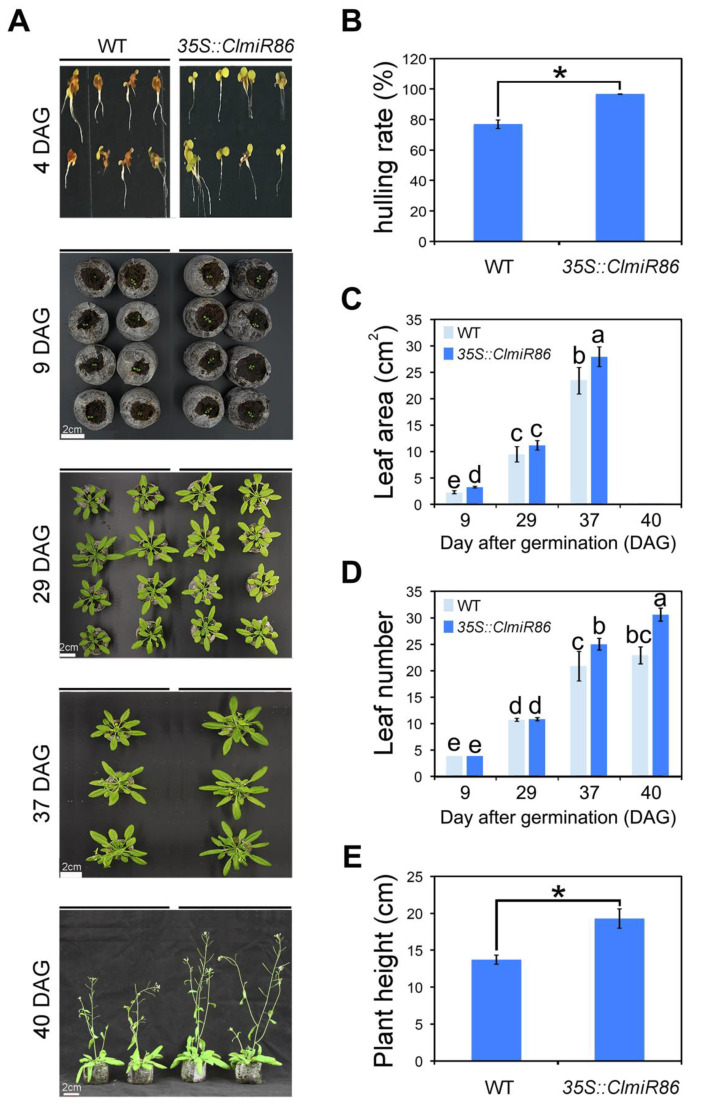
Ectopic expression of *ClmiR86* enhanced plant growth under normal conditions. (**A**) Phenotype of wild type (WT) and *35S::miR86* seedlings at 4, 9, 29, 37, and 40 day-after-germination (DAG). (**B**) Hulling rate of *Arabidopsis* seeds at 4 DAG age. (**C**) Average leaf area of *Arabidopsis* seedlings at different Age. (**D**) Average leaf number of *Arabidopsis* seedlings at different DAG age. (**E**) Plant/inflorescence height of *Arabidopsis* seedlings at 40 DAG age. Asterisk (*) or different lower-case letters indicates significant difference in hulling rate, leaf area and number, or plant height (*p* < 0.05, one-way ANOVA and then Tukey’s test for multiple comparisons). Values are means ± SD (*n* = 10).

To test whether the *ClmiR86*–*ClCIPK5* signaling axis is involved in grafting-enhanced growth and phosphate assimilation in watermelon, we examined the phenotypic differences between wildtype and transgenic *Arabidopsis* under normal conditions. Following germination on half-strength MS medium for 4 days, 35S::*ClmiR86* seeds showed a higher hulling rate than their background (Figure 7A,B), resulting in a larger leaf area in transgenic seedlings at the early stage (9 DAG) than in wildtype seedlings. However, nearly throughout the vegetative phase, no visible differences in leaf growth were observed between the transgenic and wildtype seedlings (Figure 7C). Interestingly, 35S::*ClmiR86* plants exhibited more vigorous growth than wildtype seedlings after they reached the generative phase (29–30 DAG), resulting in a larger photosynthetic area and longer inflorescence (Figure 7C,D). To establish a link between *ClmiR86*–*ClCIPK5* signaling and enhanced PUE, we analyzed the expression of 12 phosphate transporter genes (*PHTs*) using qRT-PCR. Transcripts of several of these genes were upregulated in *ClmiR86*-overexpressing lines compared with in wildtype seedlings (Figure S2B). Three CBL genes, namely *AtCBL1*, *AtCBL3,* and *AtCBL4*, interact with CIPK5 [45]. *AtCBL1* and *AtCBL4* expression was clearly increased in *ClmiR86*-overexpressing lines, perhaps due to feedback regulation (Figure S2C). These results indicate that *ClmiR86* overexpression promoted plant growth under normal conditions, which is likely related to enhanced phosphate transport via altered CIPK–CBL signaling.

## 3. Discussion

Grafting has been adopted as one of the most effective techniques for improving plant stress tolerance and modifying ion accumulation in a number of plant species [2,12,46]. It is also commonly used in horticultural crops to increase nutrient uptake and utilization, which reduces not only crop production costs, but also global environmental pollution. Here, we demonstrated the possible role of *ClmiR86*, a novel *Cucurbitaceae* miRNA, as a positive regulator of phosphate deficiency tolerance and grafting-enhanced phosphate utilization in watermelon.

### 3.1. Squash Grafting Enhanced Watermelon Phosphate Utilization

Watermelon is one of the most important economic crops in the world. Squash is commonly used as a rootstock for watermelon grafting, leading to greater production and stronger resistance/tolerance to various stresses. In several studies, squash grafting was found to increase iron, nitrate, potassium, calcium, or magnesium ion concentration in watermelon [8,10,11,12]. In addition, we previously demonstrated enhanced phosphate assimilation with squash grafting [9]. However, the effects of rootstock and scion on each other and their overall effects on nutrient absorption remain largely unknown. Typically, modification of the root architecture is a popular means of enhancing ion uptake, accumulation, and utilization efficiency. Indeed, the squash rootstock has a vigorous root system, with more lateral roots and root hair, higher total root length, and greater root surface area than watermelon. The root dry weight of pumpkin-grafted watermelon was 2.24 higher than that of self-rooted plants [47]. Some plant metabolites, including sugars, hormones, and miRNAs, may function as long-distance signaling molecules and regulate ion uptake and/or homeostasis by affecting the activity of ion transporters. Unfortunately, research on ion transporters and miRNA-regulated nutrient uptake has focused on model and non-grafted plants, and information on grafted horticultural crops is limited. In the present study, the link between squash grafting-enhanced phosphate utilization and *ClmiR86* accumulation (Figure 1; [9]) provided an important clue for elucidating the mechanism of grafting-enhanced abiotic stress tolerance and developing improved nutrient-efficient rootstocks.

### 3.2. ClmiR86 Is Involved in Phosphate Starvation Response and Phosphate Utilization in Watermelon

The roles of miRNAs in nutrient acquisition, transport, and homeostasis have been summarized in the literature [27,48]. Some miRNAs (e.g., *miR160*, *miR167*, *miR393*, *miR171*, and *miR3979*) have been proposed to play roles in nitrate uptake, possibly by promoting or suppressing root functions (primary root elongation and lateral or adventitious root emergence) [49,50,51,52]. *miR169*, *miR156*, and *miR826*/*miR5090* affect nitrate uptake, metabolism, and consumption [53,54,55]. In addition, *miR444*, which targets *MADS23*, may be involved in nitrate and potassium uptake or translocation from old to young leaves [27,56]. Recently, *miR399* and *miR827* were demonstrated to be important for the phosphate starvation response, as they inhibit genes involved in *PHT1* and *PHO1* suppression, contributing to their accumulation under stress [25,26,27]. *PHT1* proteins can use energy to cotransport Pi and H^+^ ions and are therefore involved in inorganic phosphate acquisition [57]. *PHO1* is involved in the loading of the acquired inorganic phosphate into the xylem and facilitating the root-to-shoot transport of this macronutrient in plants [58,59]. The *miR399*–*PHO2* and *miR827*–*NLA* pathways are likely coordinated to allow for fine-tuning of the expression of the high-affinity Pi transporter *PHT1* for maintaining phosphate homeostasis [27,60,61,62]. Here, we reported the novel watermelon miRNA *ClmiR86*, which was induced by grafting and phosphate deficiency (Figure 1 and Figure 2). Ectopic expression of this miRNA in *Arabidopsis* enhanced phosphate deficiency tolerance based on its promoting effect on primary root elongation and lateral root emergence (Figure 5). *miR86* function may be conserved between watermelon and *Arabidopsis*, as mature *miR86* was also detected in wildtype *Arabidopsis* plants (Figure S2A). Interestingly, this root phenotype was similar to that of the phosphate-over-accumulating mutant *pho2* grown under the same conditions (Figure S1), suggesting that *ClmiR86*-triggered phosphate deficiency tolerance is due to enhanced phosphate assimilation. In addition, the expression of several phosphate acquisition-related genes was affected in transgenic *Arabidopsis* seedlings (Figure S2B), similar to that in the *pho2* mutant [62], suggesting that *ClmiR86* is involved in phosphate signaling via the functions of these genes.

Squash grafting enhanced *ClmiR86* accumulation in scion leaves compared with auto-grafting (Figure 1A), which is consistent with the results observed by Liu et al. [1] that grafting altered miRNA expression levels in the leaves of pumpkin-grafted watermelon seedlings. In addition, our findings implied that *ClmiR86* might act as a graft-transmissible signal exported from the squash rootstock to watermelon leaves or reverse responses to both squash grafting and phosphate deficiency in leaves and roots (Figure 1). Similarly, Li et al. [63] showed that the expression of some miRNAs in the leaves and roots differed between pumpkin- and auto-grafted cucumbers as well as between seedlings grown under nitrate and phosphate deficiency. Among these, the expression levels of *miR399* and *csa-miR-n08* were increased in leaves, but decreased in the roots of pumpkin-grafted cucumber seedlings compared with those of auto-grafted seedlings. The altered miRNA levels then affected the expression of MYB transcription factors, including *phosphate starvation regulator 1* (*PHR1*), *phosphate starvation regulator 1-like* (*PHR1-LIKE1*), and *E3* (ubiquitin–protein ligase) in cucumber, which are essential for phosphate absorption [63]. Similar response patterns of *ClmiR86* and *csa-miR-n08* to both grafting and phosphate deficiency in scion and rootstock indicate shared or conserved regulatory network in squash-grafted watermelon and cucumber under phosphate starvation.

### 3.3. CIPK5 Works Downstream of ClmiR86 under Phosphate Starvation and Normal Conditions

In plants, the CBL–CIPK signal components form a complex signaling network, which allows for flexible but specific signal–response coupling during adaptation to environmental stresses and nutrient deficiency [64]. The CBL–CIPK pathways have been reported to function as nutrient transport and homeostasis regulatory networks in response to low potassium [65,66], sodium [41,42], magnesium [67], nitrate [43,68], and phosphate [36]. In the present study, watermelon *CIPK5* was found to be involved in early phosphate signaling, and *ClCIPK5* transcript levels were rapidly decreased by squash grafting (Figure 3). In addition, *ClmiR86* overexpression significantly decreased *AtCIPK5* and *AtCIPK25* (orthologs of *ClCIPK5*) expression, perhaps due to enhanced plant tolerance to phosphate deficiency (Figure 4, Figure 5 and Figure 6). Recently, *Brassica napus* CIPK (*BnCIPK6*) was also found to be involved in the phosphate starvation response. *BnCIPK6* expression was strongly induced by phosphate deficiency, and transgenic *Arabidopsis* seedlings overexpressing *BnCIPK6* grew better than the wildtype ones under phosphate starvation [36]. These results suggest a positive function of CIPK in phosphate deficiency tolerance, contrary to that of *ClCIPK5*. Orthologous genes of *BnCIPK6* in *Arabidopsis* and chickpea (*AtCIPK6* of *CaCIPK6*) were found to affect lateral root formation and salinity tolerance, possibly via their function in auxin transport and sensitivity, highlighting the role of CIPKs in root development [69]. This is partly consistent with our findings that reduced *ClCIPK5* transcript levels in transgenic *Arabidopsis* may result in significant lateral root differences (Figure 5 and Figure 6). A recent study indicated that the CIPK23–CBL9 complex could regulate primary nitrate responses via its phosphorylation function on the dual-affinity nitrate transporter CHL1 [43]. Of note, even though no functional analysis of *CIPK5* or *CIPK25* has been performed in *Arabidopsis*, CIPK5 was found to weakly interact with CBL1 in a yeast two-hybrid system [70]. This finding indicates a possible indirect interaction between CIPK5 and CIPK23 during complex formation as well as a cross-talk between nitrate and phosphate starvation responses. Unfortunately, the downstream substrates of *CIPK6*, *CIPK5*, or *CIPK25*, which might directly affect phosphate sensing or transport, have not been identified. Knowledge of these substrates will become a critical piece for solving the jigsaw puzzle of plant phosphate starvation responses. Thus, the CIPK5 downstream substrates mediating interaction with CBL must be identified to unveil the mechanism of grafting-triggered phosphate deficiency tolerance and phosphate assimilation.

In conclusion, we identified a novel grafting-induced or grafting-transportable miRNA in watermelon. *ClmiR86* likely targets the transcripts encoding CIPK5 and CIPK25, which are involved in phosphorus sensing or stress signaling. Plants overexpressing *ClmiR86* showed decreased expression of the target *CIPK* genes, enhanced lateral root growth under phosphate deficiency, and increased growth vigor under normal conditions. These findings provide valuable clues to further explore the mechanism of grafting-enhanced stress tolerance and nutrient utilization.

## 4. Materials and Methods

### 4.1. Plant Materials, Growth Conditions, and Treatments

The watermelon cultivar ‘Zaojia’ (ZJ) was grafted onto the squash cultivar ‘Feichangfuzuo’ (Wm/Sq) and watermelon (Wm/Wm). Self-rooted squash plants were used as controls for squash roots. Two watermelon genotypes, namely ‘Xinong 8 Hao’ (XN8) and ZJ, with different PUE were used to characterize the genotypic variations in *ClmiR86* and *ClCIPK5* expression [9]. Following seed germination, all watermelon and squash seedlings were grown in the Professional Growing Mix (Fafard^®^ 51 L Mix). Grafting was performed when the scion and rootstocks were at the cotyledon stage and one-leaf-stage, respectively. All plants were cultivated in a growth chamber under photosynthetically active radiation at 28 °C (day)/24 °C (night) under a 16 h light period at an intensity of 600 µmol·m^−2^·s^−1^ and 50–85% humidity. Phosphate starvation treatments were performed until the grafted plants and the two watermelon genotypes reached the two-true-leaf stage.

Under phosphate deficiency stress, all watermelon seedlings were transferred to hydroponic growth containers with full-strength Hoagland solution (0.3 mM phosphate; PH 6) and acclimated for 3 days. The seedlings were then subjected to phosphate starvation (0.01 mM) (Table S1). After 6 h of treatment, the grafted watermelon leaves and squash roots were collected. Root samples were also harvested from XN8 and ZJ at specific time intervals (6, 12, and 24 h). All samples were stored at −80 °C for further assays.

Seeds of *Arabidopsis pho2* mutants were kindly provided by Professor Huixia Shou (Zhejiang university, China) [71]. *Arabidopsis thaliana* ecotype Columbia-0 (WT) was used to generate *ClmiR86* overexpression lines (35S::*ClmiR86*). For growth and development studies, WT and 35S::*ClmiR86 Arabidopsis* seeds were sown on the Jiffy seedling culture substrate, stratified at 4 °C for 3 days and then grown in growth chambers (Sanyo; http://www.sanyobiomedical.co.uk, accessed on 3 September 2021) at 22 °C under a 16 h photoperiod at 200 µmol·m^−2^·s^−1^ light intensity and 60% relative humidity. Rosette leaf samples were collected and stored at −80 °C for further assays.

For the *Arabidopsis* phosphate starvation experiment at the seedling stage, WT and 35S::*ClmiR86*, *pho2* mutant seeds were surface sterilized twice with 20% (*v*/*v*) bleach for 1 min. After washing three times with sterile distilled water, the seeds were cultured in phosphate-sufficient (P^+^) medium containing half-strength MS medium (PhytoTechnology Laboratories), 1% (*w*/*v*) sucrose, and 1.2% (*w*/*v*) agar; vernalized at 4 °C for 3 days; and grown vertically in a growth chamber for 1 week (Sanyo). Thereafter, some 1-week-old plants were transferred onto a phosphate-deficient (P^−^) medium, in which 1.25 mM KH_2_PO_4_ in the P^+^ medium was replaced with 0.65 mM K_2_SO_4_, while the remaining plants grown on P^+^ and half-strength MS medium as the controls (Table S2). After 1 week of culture, leaf area, primary root length, and lateral root number were measured and photographed. Six seedlings were measured for each treatment, and three biological replicates were performed.

For *Arabidopsis* hydroponic culture at the adult stage, WT and 35S::*ClmiR86 Arabidopsis* adult-plants (22-day old) were transferred to hydroponic culture with half-strength Hoagland solution containing 0.3 mM phosphate for additional 7 or 17 days. Phosphate starvation was initiated by replacing 0.3 mM phosphate with 0.01 mM phosphate (Table S1).

### 4.2. Vector Construction

To generate 35S::*ClmiR86 Arabidopsis* lines, a 272 bp fragment containing the *ClmiR86* precursor foldback structure was amplified using total RNA extracted from watermelon leaves with the primer pair Pre-miR86-F and Pre-miR86-R; cloned into pDONR221 (Invitrogen); and recombined into the GatewayTM vector pMDC83 binary expression vector under the control of the CaMV *35S* promoter using the Gateway cloning system [72]. The primers used for vector construction are shown in Table S3.

### 4.3. Arabidopsis Transformation

The *ClmiR86*-pMDC83 construct was introduced into *Agrobacterium tumefaciens* strain GV3101. Wildtype *Arabidopsis* was transformed using the floral dip method [73]. To screen for transgenic plants, seeds harvested from transformed *Arabidopsis* plants were germinated on MS medium containing 50 μg·mL^−1^ hygromycin B (Roche, http://www.roche.com; accessed on 10 September 2021 ), vernalized at 4 °C for 3 days, and incubated in a growth chamber (Sanyo) for 10 days. The 10-day-old hygromycin B-resistant plants were then transferred to the Jiffy seedling culture substrate. PCR and quantitative real-time PCR (qRT-PCR) of *ClmiR86* in candidate transgenic *Arabidopsis* plants were used to confirm the success of transformation (Figures S1 and S2A). WT and T3 plants were used in the present study. All primers used for the confirmation of *Arabidopsis* transformation are listed in Table S3.

### 4.4. Target Gene Prediction

The *ClmiR86* mature sequence was used as the query to search for putative targets against the watermelon (ICuGI; http://www.icugi.org/cgi-bin/ICuGI/index.cgi; accessed on 10 September 2021 ) and *Arabidopsis* (TAIR; http://www.arabidopsis.org; accessed on 10 September 2021 ) databases with TagetFinder 1.6. To detect whether the target sites were conserved in *Arabidopsis*, the coding sequences of *ClCIPK5*, *AtCIPK5*, and *AtCIPK25* in the genomes of watermelon and *Arabidopsis* were retrieved from the ICuGI and TAIR databases, respectively. A multiple sequence alignment of the nucleotide sequences of CIPKs (*ClCIPK5*, *AtCIPK5*, and *AtCIPK25*) and *ClmiR86* was performed using ClustalX 2.0 with default settings, as described by Thompson et al. [74].

### 4.5. Phylogenetic Tree Construction

To identify the orthologous genes of *ClCIPK5* in *Arabidopsis*, the amino acid sequences of 26 CIPKs in the genome of *Arabidopsis* were retrieved from the TAIR database (http://www.arabidopsis.org; accessed on 10 September 2021 ). A neighbor–joining tree based on the protein sequence alignments was constructed using MEGA 6.0, with 1000 bootstrap replicates.

### 4.6. PUE Measurement

Shoots of the WT and *35S::ClmiR86* seedlings grown in either P^+^ or P^−^ medium or soil were sampled separately. Phosphate levels were measured using the vanadium–molybdenum method [75]. PUE was calculated as plant total dry weight divided by phosphate levels [76].

### 4.7. RNA Isolation, cDNA Synthesis, and qRT-PCR

Total RNA, including miRNA, was extracted using the mirVana^TM^ miRNA Isolation Kit (Ambion). RNA quantity and quality were assessed spectrophotometrically using the Thermo 2000 Bioanalyzer with RNA NanoDrop (Thermo Scientific; http://www.thermo.com; accessed on 10 September 2021 ). Samples showing A260/A230 ratios of 2.0–2.2 and A260/A280 ratios of 1.8–2.0 were used for further analysis. For quantitative real-time PCR, 1 µg of total RNA was reverse transcribed to first-strand cDNA in a final reaction volume of 20 µL using the miScript II RT Kit (Qiagen).

The *ClmiR86* forward primer was designed based on its mature sequence, and the universal reverse primer was provided with the miScript SYBR Green PCR Kit (Qiagen). *miR167c* was used as a reference gene to normalize *miR86* expression in watermelon and squash [77]. Primers specific to the *ClmiR86* precursor were used to detect Pre-miR86 expression levels. Pre-miR86 and *ClCIPK5* expression in watermelon was normalized to yellow-leaf-specific protein 8 (*ClYLS8*) expression. *AtUBQ10* was selected as a reference gene for *Arabidopsis* gene expression [25]. All primer sequences are listed in Table S4.

qRT-PCR was performed on the StepOnePlus™ Real-Time PCR System (ABI) using the miScript SYBR Green PCR Kit (Qiagen). PCR included pre-incubation at 95 °C for 15 min, followed by 40 cycles of denaturation at 94 °C for 15 s, annealing at 55 °C for 30 s, and extension at 70 °C for 30 s. Primer amplification specificity and efficiency were measured by melt curve and standard curve analysis, respectively. The relative expression levels were calculated using the 2^−∆∆CT^ method. Each experiment was performed in triplicate.

### 4.8. Statistical Analysis

For the statistical analysis, one-way ANOVA followed by Tukey post hoc test were performed using SPSS Statistics Version 22.

## Supplementary Materials

The following are available online at https://www.mdpi.com/article/10.3390/plants10102133/s1, Figure S1. Root phenotypes of *pho2* mutant under P starvation condition. Figure S2. Expression of *CIPK, CBL* and *PHT* genes in transgenic and wild type *Arabidopsis.* Figure S3. PCR confirmation of *ClmiR86* transformation in candidate transgenic Arabidopsis. Table S1. Formula of Hoagland nutrient solution. Table S2. Formula of full-strength MS medium. Table S3. Primers used for vector construction and confirmation of *Arabidopsis* transformation. Table S4. Gene-specific primers for qRT-PCR.
