# Supplementary figures and images for: The Novel Cucurbitaceae miRNA ClmiR86 Is Involved in Grafting-Enhanced Phosphate Utilization and Phosphate Starvation Tolerance in Watermelon
Source: Plants (Basel). 2021 Oct 8;10(10):2133. doi: 10.3390/plants10102133 (PMC8540214; doi:10.3390/plants10102133)

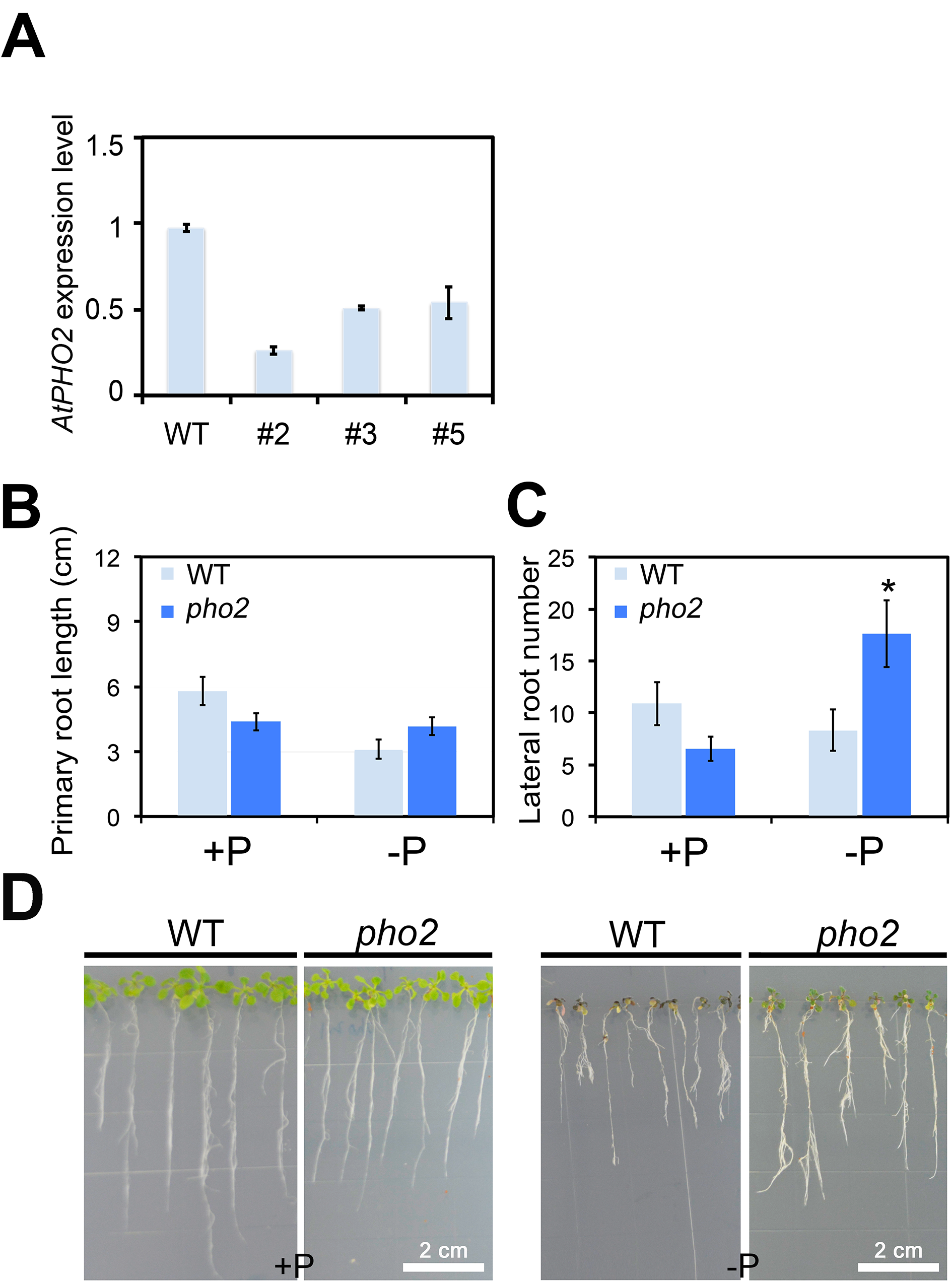

Supplement: Supplementary file 1 [file plants-10-02133-s001.zip › FigS1-20211008.tif]

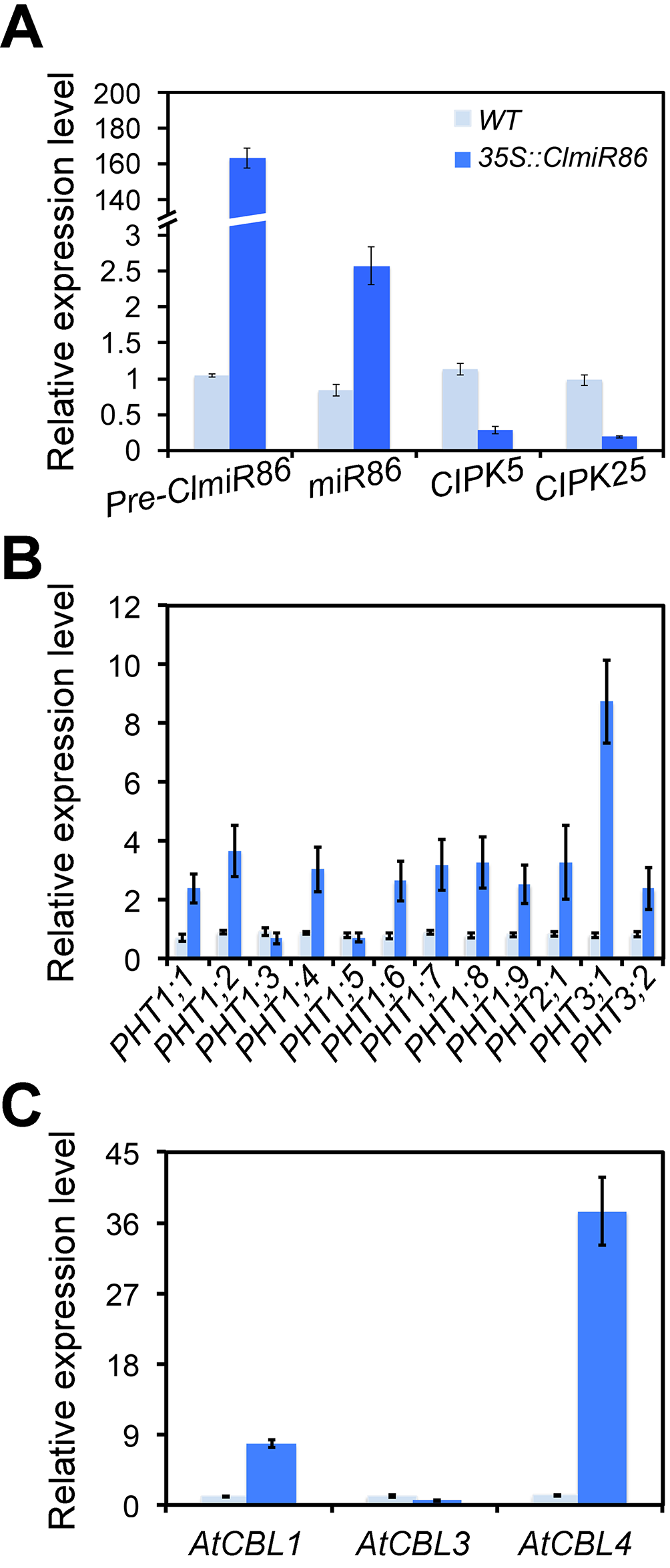

Supplement: Supplementary file 1 [file plants-10-02133-s001.zip › FigS2-20210308.tif]

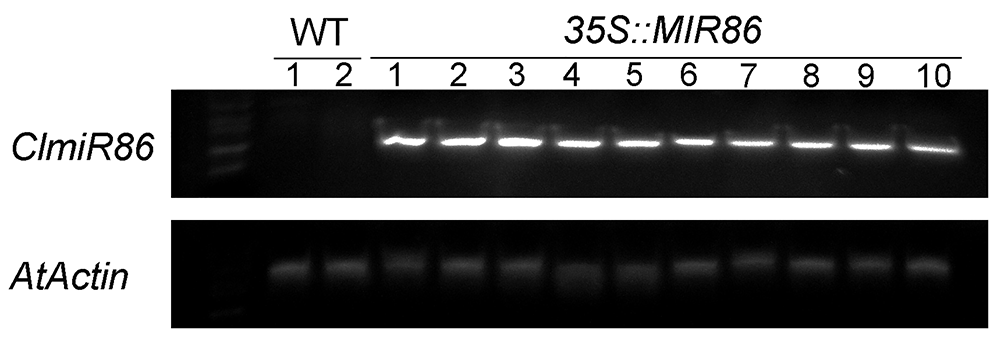

Supplement: Supplementary file 1 [file plants-10-02133-s001.zip › FigS3-20211008.tif]
